# Annotated checklist of the coastal ichthyofauna from Michoacán State, Mexico

**DOI:** 10.3897/zookeys.606.9004

**Published:** 2016-07-21

**Authors:** Eloísa Torres-Hernández, Georgina Palacios-Morales, Salvador Romero-Gallardo, Paloma Salazar-Araujo, Adrián García-Meraz, Xavier Madrigal-Guridi, Luis F. Del Moral-Flores, Omar Domínguez-Domínguez

**Affiliations:** 1 Laboratorio de Biología Acuática Facultad de Biología Universidad Michoacana de San Nicolás de Hidalgo Ciudad Universitaria s/n Morelia Michoacán México; 2 Posgrado de Ciencias del Mar y Limnología Unidad Académica Mazatlán Universidad Nacional Autónoma de México Apartado postal 811 Mazatlán 82000 Sinaloa México; 3 Laboratorio de Zoología, Facultad de Estudios Superiores Iztacala, Universidad Nacional Autónoma de México, 54090 Tlalnepantla, Estado de México, México.

**Keywords:** Coastal fish, estuaries, marine, Mexican Central Pacific, systematic list

## Abstract

This study is the first to complete an intensive and comprehensive list of the ichthyofauna of nearly all ecosystems of the Michoacán coast, Mexico. The resulting systematic checklist, supplemented with information from the literature and scientific collections, comprises 440 species belonging to two classes, 31 orders, 104 families, and 264 genera. The families with the highest number of species were Sciaenidae (30 spp.), Carangidae (26), Haemulidae (24), Serranidae (21), Paralichthyidae, and Gobiidae (13). Of the total species list, 134 represent first records for the Michoacán State, and one is a first record for Mexico. The results expand the number of known fish species of the Michoacán coast by almost one third and will help to develop conservation and management plans for this coastal zone.

## Introduction

Mexico has a wealth of both marine species and ecosystems. The country has the world’s twelfth longest marine territory, including both Atlantic and Pacific oceans. The geographic and geological history, as well as the ecological and biological richness of areas such as the Gulf of California, the Oceanic islands, and the Caribbean, expands the diversity of Mexican marine life (Lara-Lara et al. 2008), including the ichthyofauna. The few studies pertaining to these taxa have primarily focused on conservation priority zones such as the Gulf of California (e.g. Del Moral-Flores et al. 2013), protected marine areas (e.g. Galván-Villa et al. 2016), or on economically important species (e.g. Lara-Lara et al. 2008).

The Mexican tropical Pacific is part of the biogeographic region known as the Tropical Eastern Pacific (TEP), which comprises three provinces: Cortez or Sinuscaliforniana, Mexican, and Panamic (*sensu* Briggs 1974). An estimated 1358 fish species occur in the TEP region, along with an additional of approximately 59 undescribed species (Zapata and Robertson, 2007). The TEP could be considered an area of low richness compared to other biogeographical regions of tropical seas, such as the Indo-Malaysian or the Great Caribbean. However, approximately 71% of identified TEP fish species are considered endemic, making it the tropical region with the highest rate of endemism per unit area in the world (Robertson and Allen 2015). According to Robertson and Allen (2015), the Cortez province possesses 9% of the 515 endemic fish species identified in the coastal ecosystems of the TEP region, whereas the Mexican and the Panamic provinces host 2% and 29% of the endemic component, respectively. Nine percent of the endemic species occur in both the Cortez and Mexican provinces, 10% in both the Mexican and the Panamic provinces, and 37% of the species are found in the three provinces. The endemic fish fauna of the five TEP oceanic islands (Revillagigedo, Galapagos, Cliperton, Coco, and Malpelo) represent, on the other hand, 10% of the total.

The Mexican province is highly productive due to the convergence of the Costa Rica Coastal Current and the California Current, favoring the presence of tropical, temperate, and transitional fish species (Kessler 2006). Based on its lower number of endemic fish species, the Mexican province has been considered a transition zone between the Cortez province in the north and the Panamic to the south (Hastings 2000, Palacios-Salgado 2005, Robertson and Cramer 2009). Attempts to characterize the ichthyofauna of this province are scarce, and most of them refer to a particular group of fishes, region or are based on unpublished reports (Madrid-Vera et al. 1998; Palacios-Salgado 2005, Madrigal-Guridi 2006, Moncayo-Estrada et al. 2006, Chávez-Comparan et al. 2008, López-Pérez et al. 2010, Márquez-Espinoza 2012, Sandoval-Huerta et al. 2012, 2012b, 2014; Palacios-Morales et al. 2014).

The coastline of the Michoacán State, in the Mexican province, is 261.5 km in length and runs from Boca de Apiza, at the mouth of the Coahuayana River, which represents the border with the Colima State to the north, to Barra de San Francisco, at the mouth of the Balsas River, which represents the border with the Guerrero State to the south (Correa and Gómez 2003). There are two contrasting physiographic zones differing markedly in the marine ecosystems and consequently in the fish species present: (1) the municipalities of Lazaro Cardenas and Coahuayana, which are characterized by coastal plains, with a wide sandy coastline, mangroves, and estuary zones, (2) and the municipality of Aquila comprising numerous cliffs extending into the sea and forming wide zones of rocky reefs, coralline patches, and intertidal pools; estuaries in the last zone are scarce and differ in size and dynamism from those found in Lazaro Cardenas and Coahuyana (Correa and Gómez 2003).

Such heterogeneity in a transitional zone potentially produces high fish species richness. Nevertheless, information on the ichthyofauna of the Michoacán coast is limited, including two study focused on artisanal fishery species (Amezcua-Linares 2009, Sánchez-Aguilar 2007), two on estuarine fishes (Madrigal-Guridi 2006, Sandoval-Huerta et al. 2014) and one including all habitats (Medina-Nava et al. 2001). Madrid-Vera et al. (1998) published the previously most extensive list of the fish fauna of the Michoacán coast, with 257 species, 157 genera, and 76 families recorded in a wide variety of environments. This limited knowledge of the fish fauna contrasts with the importance of the fishery to the economy of the region as the main economic activity, with about 11,931 fishermen producing 6525 tons with an estimated economic value of 145,255,860 MXN (CONAPESCA 2014).

The main goal of this study was to provide an updated checklist of the ichthyofauna from the Michoacán coast including information on fish of local commercial importance and their biogeographic affinity. This knowledge will increase the understanding of regional fish diversity and could be of usefulness for conservation and management strategies of the littoral zone of the Central Mexican Pacific and particularly for the Michoacán State.

## Materials and methods

**Figure 1.**
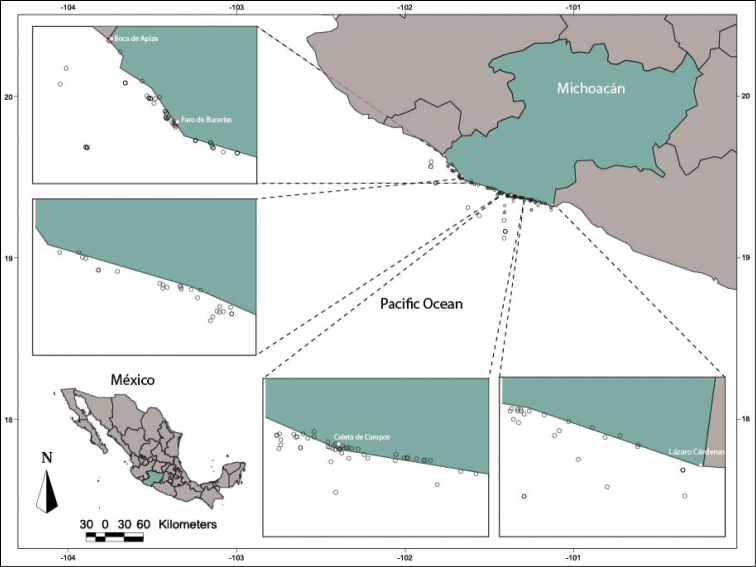
Sampling locations on the coast of Michoacán State.

The study area encompassed the coastline of Michoacán state, with 110 locations directly sampled (Fig. 1) and information on 50 additional sites obtained from published literature or scientific collections. These data were obtained through extensive review of the biological material deposited at the Colección de Peces de la Universidad Michoacana de San Nicolás de Hidalgo
(CPUM), the Colección Nacional de Peces (UNAM), the Colección Ictiológica del Instituto de Ciencias del Mar y Limnología (UNAM) and the Marine Vertebrate Collection, Scripps Institute of Oceanography (**SIO**). In addition, records from the data base of the fish collection of the California Academy of Sciences
(CAS) were reviewed. These investigation also included an extensive review of specialized publications (books, catalogues, and field guides) and reports of specimens deposited in ichthyological collections recognized by the Secretaria de Medio Ambiente y Recursos Naturales, México, or specimens of which identification was corroborated by experts.

Field sampling was conducted bimonthly from February 2010 to February 2011, with intermittent sampling in the ensuing year. Sampling was carried out in estuarine zones, rocky intertidal pools, rocky reefs, sandy areas, coralline communities, artificial reefs, and the demersal-pelagic area. Methods were tailored to the ecosystem. Reef species were collected via SCUBA diving using elastic band harpoons. Ecologically cryptic (*sensu* Viesca-Lobatón 2005) and intertidal pool species were collected using eugenol (clove oil) anesthetic at a ratio of 1:5 (eugenol:ethanol) for reef and 0.25:9.75 for intertidal pool species. When the organisms were sedated, they were captured with a slurp gun or hand net. For estuarine locations, nocturnal sampling was done using gill nets (12 × 1.8 m and 0.7 to 1.2 cm mesh) and cast nets. For sandy-bottomed sites, a small fishing net (2 × 1.8 m, 1 cm mesh and 3 m bag) was used using a dragging period of 20 min. Captures from a shrimp fishing boat were also analyzed. Aggregations of debris in the open sea were investigated to collect ocean species rarely found in coastal areas. Cooperation with local artisanal fishermen was established to review incidental and commercial catches. Fishermen used lines of 50, 100, and 200 m at 5, 20, and 40 m depth, with hooks of various sizes, fishing with fishhook at a maximum depth of 80 m and gillnets of 7 to 12 cm mesh size. Information about the commercial value and uses of the species also was recorded.

Most specimens were photographed upon collection; tissue samples were taken and deposited at the tissue collection of the CPUM. Specimens were fixed in 5% or 10% formalin neutralized with sodium borate and posteriorly preserved in 70% ethanol. Fishes were identified using the keys and descriptions from Springer (1962), Allen and Robertson (1991, 1992, 1998), Fisher et al. (1995), Castro-Aguirre et al. (1999), Hastings and Robertson (1999, 1999b), Thomson et al. (2000), Carpenter and Niem (2001), Miller and Stefanni (2001), Miller et al. (2005), and Robertson and Allen (2015). For some groups, specialized literature was required: Balistidae (Latreille 1804, Shaw 1804-1805, Jordan and Evermann 1900, Froese and Pauly 2003), Rhinobatidae (Himaya and Kumada 1940), Gerreidae (Benitez 2004), Atherinopsidae (Lavenberg and Chernoff 1995), Labrisomidae (Hubbs 1953; Springer1959, Rosenblatt and Parr 1969, Rosenblatt and Taylor 1971), Blenniidae (Springer 1962), Chaenopsidae (Hastings and Robertson 1999), Tripterygiidae (Allen and Robertson 1991, 1992, Rosenblatt et al. 2013), and for the genera *Abudefduf* Forsskål, 1775 (Lessios et al. 1995), *Tomicodon* Brisout de Barneville, 1846, *Gobiesox* Lacepède, 1800 (Briggs 1955; Briggs and Miller 1960), and *Albula* Scopoli, 1777 (Pfeiler 2008). All specimens were deposited at CPUM (MICH-PEC-227-07-09).

The systematic arrangement followed Nelson et al. (2016). The current taxonomic status of each species was corroborated in Eschmeyer et al. (2016). The arrangement of the genera and species was in alphabetical order. In the systematic list, the habitat-type from which each species was collected and the scientific collection by which the specimen was identified, or the scientific document from which information of the specimen was obtained, are indicated.

Finally, a zoogeographical affinity analysis of the species, based on the biogeographical regionalization proposed by Briggs (1974, 1995), was made. Accordingly, the Tropical Eastern Pacific was divided into three provinces. The San Diegan province was also included, since some species tended to have a northern distribution.

## Results

Sampling was performed in 13 intertidal pool sites, 20 rocky reef sites, three coralline communities, two artificial reefs, 22 estuarine ecosystems, and 50 soft bottom and open sea sites, collecting 6963 fishes.

The compiled systematic list of ichthyofauna of the Michoacán coast comprises 436 species belonging to two classes, 31 orders, 104 families, and 260 genera (Table 1). The families representing the greatest number of species were Sciaenidae (30), Carangidae (26), Haemulidae (24), Serranidae (21), and Paralichthyidae and Gobiidae (13). The genera with the highest number of species were *Lutjanus* Bloch, 1790 (9), *Carcharhinus* Blainville, 1816 (7), *Anchoa* Jordan and Evermann, 1927 (6), *Diplectrum* Holbrook, 1855 (6), and *Caranx* Lacepède, 1801 (5).

**Table 1.** Updated checklist of the coastal ichthyofauna from Michoacán, Mexico.

	Collected habitat (2010–2011)	Ichthyogeographic affinity	References and organisms voucher	The importance in fisheries
**CLASS ELASMOBRANCHII**				
**ORDER ORECTOLOBIFORMES**				
**FAMILY GINGLYMOSTOMATIDAE**				
*Ginglymostoma unami* Del Moral Flores, Ramírez-Antonio, Angulo y Pérez-Ponce de León, 2015	R	AA	2, 4, 6, CPUM	A
**ORDER LAMNIFORMES**				
**FAMILY LAMINIDAE**				
*Isurus oxyrinchus* Rafinesque, 1810		CT	1	
**FAMILY ALOPIIDAE**				
*Alopias pelagicus* Nakamura, 1935		AP	2	
*Alopias superciliosus* (Lowe, 1841)		CT	2	
**ORDER CARCHARHINIFORMES**				
**FAMILY TRIAKIDAE**				
*Mustelus lunulatus* Jordan & Gilbert, 1882	D	TEP	2, 6	
**FAMILY CARCHARHINIDAE**				
*Carcharhinus albimarginatus* (Rüppell, 1837)		CT	2	
*Carcharhinus brachyurus* (Günther, 1870)		CT	2	
*Carcharhinus cerdale* Gilbert, 1898		TEP	1, 2	
*Carcharhinus falciformis* (Müller & Henle, 1839)		CT	2	
*Carcharhinus leucas* (Müller & Henle, 1839)		CT	1, 2	
*Carcharhinus limbatus* (Müller & Henle, 1839)	D	CT	1, 2, 6, CPUM	C
*Carcharhinus obscurus* (Lesueur, 1818)		CT	1, 2	
*Galeocerdo cuvier* (Péron & Lesueur, 1822)		CT	1, 2	
*Nasolamia velox* (Gilbert, 1898)		TEP	2	
*Negaprion brevirostris* (Poey, 1868)		AA	1, 2	
*Rhizoprionodon longurio* (Jordan & Gilbert, 1882)	D	SP, TEP	1, 2, CPUM	C
**FAMILY SPHYRNIDAE**				
*Sphyrna lewini* (Griffith & Smith, 1834)	D	CT	1, 2, 4, 6, 9, CPUM	C
*Sphyrna zygaena* (Linnaeus, 1758)		CT	2	
**ORDER TORPEDINIFORMES**				
**FAMILY NARCINIDAE**				
*Narcine entemedor* Jordan & Starks, 1895	D	TEP	1, 9, CPUM	I
*Narcine vermiculatus Breder, 1928	D	MP, PP	6, 9, CPUM, ICMYL, CIBNOR	I
**ORDER PRISTIFORMES**				
**FAMILY PRISTIDAE**				
*Pristis pristis (Linnaeus, 1758)	PD	AA	CPUM-photo	C
**ORDER RAJIFORMES**				
**FAMILY RHINOBATIDAE**				
*Rhinobatos glaucostigma* Jordan & Gilbert, 1883	R	TEP	1, 2, 4, 6, 9, CPUM, ICMYL	C
*Rhinobatos productus* Ayres, 1856		SP, TEP	2	
*Zapteryx xyster Jordan & Evermann,1896	R	TEP	CPUM, SIO	I
**FAMILY RAJIDAE**				
*Raja equatorialis* Jordan & Bollman, 1890		MP, PP	1	
**ORDER MYLIOBATIFORMES**				
**FAMILY UROTRYGONIDAE**				
Urotrygon aff. aspidura (Jordan & Gilbert, 1882)		TEP	1	
*Urotrygon chilensis* (Günther, 1872)		TEP	1, 2, 9	
*Urotrygon munda* Gill, 1863		TEP	2	
*Urotrygon nana* Miyake & McEachran, 1988		TEP	1, 9	
*Urotrygon rogersi* (Jordan & Starks, 1895)	PD	TEP	1, 4, 9, CPUM, SIO	I
**FAMILY GYMNURIDAE**				
*Gymnura marmorata* (Cooper, 1863)		SP, TEP	1, 9, CPUM	C
**FAMILY MYLIOBATIDAE**				
*Aetobatus laticeps* (Euphrasen, 1790)	R	CT	2, 4, CPUM-photo	I
**FAMILY MYLIOBATIDAE**				
*Rhinoptera steindachneri Evermann & Jenkins, 1891	PD	TEP	CPUM	I
**FAMILY MOBULIDAE**				
*Mobula munkiana Notarbartolo di Sciara, 1987	PD	TEP	CPUM	C
**FAMILY UROTRYGONIDAE**				
*Urobatis concentricus* Osburn & Nichols, 1916	R	TEP	1, 6, CPUM	
*Urobatis halleri* (Cooper, 1863)	R	TEP	2, CPUM	
**FAMILY DASYATIDAE**				
*Dasyatis dipterura* (Jordan & Gilbert, 1880)	D	TEP	2, 9, CIBNOR	
*Dasyatis longus* (Garman, 1880)	PD	SP, TEP	1, CPUM	I
**CLASS ACTINOPTERYGII**				
**ORDER ELOPIFORMES**				
**FAMILY ELOPIDAE**				
*Elops affinis* Regan, 1909	E	SP, TEP	2, CPUM	C
**ORDER ALBULIFORMES**				
**FAMILY ALBULIDAE**				
*Albula pacifica* (Beebe, 1942)	D	MP, PP	1, 2, 9, CPUM	C
**ORDER ANGUILLIFORMES**				
**FAMILY MURAENIDAE**				
*Echidna nocturna (Cope, 1872)	PM, R	CP, MP	CPUM	
*Enchelycore octaviana (Myers & Wade, 1941)	PM, R	CP, MP	CPUM	
*Gymnomuraena zebra* (Shaw, 1797)	R	AP	2, 6, CPUM	I
*Gymnothorax castaneus* (Jordan & Gilbert, 1883)	PM, R	TEP	2, 6, CPUM	
*Gymnothorax equatorialis (Hildebrand, 1946)	R	TEP	9, CPUM	
*Muraena argus (Steindachner, 1870)		TEP	ICMYL	
*Muraena lentiginosa* Jenyns, 1842	PM, R	TEP	2, 4, 5, 9, CPUM	
*Uropterygius macrocephalus (Bleeker, 1864)	PM	TEP	CPUM	
**FAMILY OPHICHTHIDAE**				
*Apterichtus equatorialis (Myers & Wade, 1941)	R	TEP	CPUM	
*Echiophis brunneus (Castro-Aguirre & Suárez de los Cobos, 1983)	R	TEP	9, CPUM	
*Myrichthys aspetocheiros McCosker & Rosenblatt, 1993	R	CP, MP	6, CPUM	
*Ophichthus triserialis* (Kaup, 1856)	R	TEP	2, CPUM, ICMYL	I
*Ophichthus zophochir* Jordan & Gilbert, 1882	R	TEP	1, 2, CPUM, ICMYL	I
**FAMILY CONGRIDAE**				
*Ariosoma gilberti (Ogilby, 1898)	D	MP, PP	9	
*Heteroconger digueti* (Pellegrin, 1923)		TEP	2	
*Paraconger californiensis Kanazawa, 1961		TEP	SIO	
*Rhynchoconger nitens (Jordan & Bollman, 1890)	D	TEP	9, CPUM, ICMYL	I
**ORDER CLUPEIFORMES**				
**FAMILY CLUPEIDAE**				
*Harengula thrissina* (Jordan & Gilbert, 1882)	PM	SP, TEP	2, 4, 6, 9, CPUM	A
*Lile gracilis* Castro-Aguirre & Vivero, 1990	E	MP, PP	1, 12, CPUM	
*Lile nigrofasciata* Castro-Aguirre, Ruiz-Campos & Balart, 2002	E	TEP	1, 10, 12, 13, CPUM	
*Lile stolifera* (Jordan & Gilbert, 1882)	E	TEP	CPUM	
*Opisthonema bulleri (Regan, 1904)	PD	TEP	CPUM	A
*Opisthonema libertate* (Günther, 1867)	E	SP, TEP	2, 8, CPUM	A
*Opisthonema medirastre Berry & Barrett, 1963	PM	TEP	CPUM	A
**FAMILY ENGRAULIDAE**				
*Anchoa argentivittata (Regan, 1904)	PM	TEP	CPUM	A
*Anchoa ischana* (Jordan & Gilbert, 1882)		TEP	2, 5	
*Anchoa lucida* (Jordan & Gilbert, 1882)	E	TEP	2, 12, CPUM	
*Anchoa mundeola* (Gilbert & Pierson, 1898)		TEP	2	
*Anchoa nasus (Kner & Steindachner, 1867)	PM	TEP	CPUM, ICMYL	
*Anchoa scofieldi* (Jordan & Culver, 1895)	PD	MP, PP	2, 4, CPUM	A
*Anchovia macrolepidota (Kner, 1863)	PD	SP, TEP	CPUM	A
*Cetengraulis mysticetus* (Günther, 1867)	PD	TEP	2, CPUM	A
**FAMILY PRISTIGASTERIDAE**				
*Ilisha fuerthii* (Steindachner, 1875)		MP, PP	2, 7	
*Opisthopterus dovii (Günther, 1868)		TEP	8	
*Pliosteostoma lutipinnis* (Jordan & Gilbert, 1882)	E	TEP	1, 8, 9, 10, 12, 13, CPUM	A
**ORDER GONORYNCHIFORMES**				
**FAMILY CHANIDAE**				
*Chanos chanos* (Forsskål, 1775)	PD	CT	2, 7, CPUM	C
**ORDER CYPRINIFORMES**				
**FAMILY CYPRINIDAE**				
*Cyprinus carpio* Linnaeus, 1758	E	Introduced	CPUM	
**ORDER SILURIFORMES**				
**FAMILY ARIIDAE**				
*Bagre panamensis* (Gill, 1863)		TEP	2	
*Bagre pinnimaculatus* (Steindachner, 1876)		TEP	2	
*Cathorops dasycephalus* (Günther, 1864)		MP, PP	2	
*Notarius kessleri* (Steindachner, 1877)		MP, PP	1, 2	
*Notarius planiceps* (Parr, 1931)		MP, PP	1, 2	
*Occidentarius platypogon* (Steindachner, 1877)	E, D	SP, TEP	1, 8, 9, CPUM	C
*Sciades guatemalensis* (Günther, 1864)	E	MP, PP	1, 2, CPUM	C
*Sciades seemanni* (Günther, 1864)		TEP	1, CNPE-IBUNAM	
**FAMILY LORICARIIDAE**				
*Pterygoplichthys disjunctivus* (Weber, 1991)	E	Introduced	10, CPUM	
**ORDER OSMERIFORMES**				
**FAMILY BATHYLAGIDAE**				
*Bathylagoides nigrigenys Garman, 1899		MP, PP	SIO	
**ORDER STOMIIFORMES**				
**FAMILY GONOSTOMATIDAE**				
*Cyclothone acclinidens (Garman, 1899)		CT	SIO	
**FAMILY PHOSICHTHYIDAE**				
*Vinciguerria lucetia (Garman, 1899)		AP	SIO	
**FAMILY STOMIIDAE**				
*Bathophilus filifer Gilbert, 1890		AP	SIO	
*Idiacanthus antrostomus (Parr, 1929)		AP	SIO	
**ORDER AULOPIFORMES**				
**FAMILY SCOPELARCHIDAE**				
*Scopelarchoides nicholsi Jordan & Bollman, 1890		SP, TEP	SIO	
**FAMILY SYNODONTIDAE**				
*Synodus evermanni Gilbert, 1890	R	TEP	9, CPUM, SIO	I
*Synodus lacertinus Jordan & Gilbert, 1882	R	TEP	9, CPUM	
*Synodus scituliceps* Hildebrand, 1946		TEP	1, 2, 9, ICMYL	
*Synodus sechurae Parr, 1931	PD	TEP	CPUM	I
**ORDER MYCTOPHIFORMES**				
**FAMILY MYCTOPHIDAE**				
*Diaphus pacificus Hubbs, 1944		AP	CAS	
*Diogenichthys laternatus (Jordan & Bollman, 1890)		AP	CAS	
*Lampanyctus omostigma (Gilbert, 1890)		AP	CAS	
*Lampanyctus parvicauda Parr, 1931		AP	SIO	
*Myctophum aurolaternatum (Putnam, 1874)		AP	ANSP	
**ORDER OPHIDIIFORMES**				
**FAMILY OPHIDIIDAE**				
*Brotula clarkae* Hubbs, 1944		TEP	1	
*Lepophidium prorates (Jordan & Bollman, 1890)	D	TEP	9, SIO, ICMYL	
*Otophidium indefatigabile (Richardson, 1844)	D	TEP	9, ICMYL, CIBNOR	
**FAMILY CARAPIDAE**				
*Carapus dubius* (Putnam, 1874)		AP	1	
**ORDER BATRACHOIDIFORMES**				
**FAMILY BATRACHOIDIDAE**				
*Batrachoides waltersi Collette & Russo, 1981		MP, PP	ICMYL	
*Porichthys ephippiatus Walker y Rosenblatt, 1988		TEP	CPUM, SIO	I
*Porichthys margaritatus (Richardson, 1844)		TEP	9, CIBNOR	
**ORDER LOPHIIFORMES**				
**FAMILY LOPHIIDAE**				
*Lophiodes caulinaris* (Garman, 1899)		SP, TEP	1, 9, SIO, ICMYL	
*Lophiodes spilurus* (Garman, 1899)		TEP	1	
**FAMILY ANTENNARIIDAE**				
*Antennatus sanguineus (Gill, 1863)		SP, TEP	6	
*Antennatus strigatus (Gill, 1863)	R	TEP	6, CPUM, ICMYL	
*Fowlerichthys avalonis (Bancroft, 1834)	R	SP, TEP	6, 9, CPUM	
**FAMILY OGCOCEPHALIDAE**				
*Zalieutes elater* (Jordan y Gilbert, 1882)	PD	SP, TEP	1, 2, 9, CPUM, SIO, ICMYL	I
**ORDER GOBIESOCIFORMES**				
**FAMILY GOBIESOCIDAE**				
*Arcos erythrops (Jordan & Gilbert, 1882)	PM, R	CP, MP	CPUM	
*Gobiesox adustus Jordan & Gilbert, 1882	PM, R	TEP	CPUM	
*Gobiesox mexicanus* Briggs & Miller, 1960	E	Freshwater	CPUM	
*Tomicodon petersii (Garman, 1875)	R	MP, PP	CPUM	
*Tomicodon zebra* (Jordan & Gilbert, 1882)	PM	CP, MP	2, CPUM	
**ORDER ATHERINIFORMES**				
**FAMILY ATHERINOPSIDAE**				
*Atherinella eriarcha Jordan y Gilbert, 1882	PM	TEP	4, CPUM, CNPE-IBUNAM	
*Atherinella guatemalensis* (Günther, 1864)	E	MP, PP	1, 8, 12, CPUM, CNPE-IBUNAM	
*Atherinella panamensis* Steindachner, 1875	E	MP, PP	8, 10, 12, CPUM	
**ORDEN CYPRINODONTIFORMES**				
**FAMILIA POECILIDAE**				
*Poecilia butleri* Jordan, 1889	E	Freshwater	8, 10, 12, 13, CPUM	
**ORDER BELONIFORMES**				
**FAMILY BELONIDAE**				
*Platybelone argalus* (Lesueur, 1821)		CT	2, SIO, ANSP	
*Strongylura exilis* (Girard, 1854)		SP, TEP	2, 6	
*Tylosurus fodiator* Jordan y Gilbert, 1882	PD	TEP	2, CPUM	C
**FAMILY HEMIRAMPHIDAE**				
*Hemiramphus saltator* Gilbert & Starks, 1904	PD	TEP	1, 6, CPUM	
*Hyporhamphus naos* Banford & Collette, 2001	PD	TEP	1, CPUM	A
*Oxyporhamphus micropterus (Valenciennes, 1847)		CT	SIO	
**FAMILY EXOCOETIDAE**				
*Cheilopogon furcatus (Mitchill, 18l5)		CT	SIO	
*Cheilopogon papilio (Clark, 1936)		SP, CP, MP	SIO	
*Cypselurus callopterus* (Günther, 1866)		SP, CP, MP	1, 2, SIO	
*Exocoetus monocirrhus Richardson, 1846		AP	SIO	
*Fodiator rostratus* (Günther, 1866)		TEP	2, SIO	
*Prognichthys tringa Breder, 1928		AP	SIO	
**ORDER STEPHANOBERYCIFORMES**				
**FAMILY MELAMPHAIDAE**				
*Scopelogadus mizolepis (Günther, 1878)		CT	CAS	
**ORDER BERYCIFORMES**				
**FAMILY HOLOCENTRIDAE**				
*Sargocentron suborbitale* (Gill,1863)	PM, R	TEP	2, 4, 5, 6, CPUM, SIO, ICMYL	
*Myripristis leiognathus* Valenciennes, 1846	R	SP, TEP	2, 5, 6, 9, CPUM	
**ORDER SYNGNATHIFORMES**				
**FAMILY FISTULARIIDAE**				
*Fistularia commersonii* Rüppell, 1838	R	AP	1, 2, 6, CPUM	
*Fistularia corneta Gilbert & Starks, 1904	R	SP, TEP	CPUM	
**FAMILY SYNGNATHIDAE**				
*Doryrhamphus excisus Kaup, 1856	R	AP	CPUM	
*Hippocampus ingens* Girard, 1858	R	SP, TEP	1, 2, 6, 9, CPUM, ICMYL	
*Pseudophallus starksii* (Jordan & Culver, 1895)	E	TEP	1, 12, CPUM, CNPE-IBUNAM	
**ORDER SCORPAENIFORMES**				
**FAMILY SCORPAENIDAE**				
*Scorpaena histrio* Jenyns, 1840		TEP	2	
*Scorpaena mystes* (Jordan & Starks, 1895)	R	AA	1, 6, 9, CPUM, ICMYL	I
*Scorpaena russula* Jordan & Bollman, 1890		TEP	1, 9	
*Scorpaena sonorae Jenkins & Evermann, 1889	PD	CP, PP	CPUM	I
*Scorpaenodes xyris (Jordan & Gilbert, 1882)	R	SP, TEP	CPUM	
**FAMILY TRIGLIDAE**				
*Bellator gymnostethus (Gilbert, 1892)		TEP	9	
*Bellator loxias* (Jordan, 1897)		TEP	2	
*Bellator xenisma* (Jordan & Bollman, 1890)		TEP	1, 2, 9	
*Prionotus albirostris* Jordan & Bollman, 1890		TEP	1, 9	
*Prionotus horrens* Richardson, 1844		TEP	1	
*Prionotus ruscarius* Gilbert & Starks, 1904	R	SP, TEP	1, 2, 9, CPUM, SIO	A
*Prionotus stephanophrys* Lockington, 1881	R	SP, TEP	1, 2, 9, CPUM, ICMYL	A
**ORDER PERCIFORMES**				
**FAMILY CENTROPOMIDAE**				
*Centropomus armatus* Gill, 1863	PD	MP, PP	2, 7, 8, CPUM	C
*Centropomus medius* Günther, 1864	PD	SP, TEP	1, 2, CPUM	C
*Centropomus nigrescens* Günther, 1864	E	TEP	2, 4, 10, 12, 13, CPUM	C
*Centropomus robalito* Jordan & Gilbert, 1882	E	TEP	2, 3, CPUM	
*Centropomus viridis* Lockington, 1877	E	TEP	1, 2, 3, 7, 8, CPUM	
**FAMILY SERRANIDAE**				
*Alphestes inmaculatus* Breder, 1936	R	TEP	2, 6, CPUM	I
*Alphestes multiguttatus* (Günther, 1867)	R	TEP	2, 4, 5, 6, CPUM	I
*Cephalopholis panamensis* (Steindachner, 1877)	R	TEP	2, 4, 6, CPUM	
*Dermatolepis dermatolepis* (Boulenger, 1895)	R	TEP	2, 6, CPUM-photo	
*Diplectrum eumelum* Rosenblatt & Johnson, 1974	R	TEP	1, 9, CPUM, SIO	
*Diplectrum euryplectrum Jordan & Bollman, 1890	R	TEP	CPUM, ICMYL	
*Diplectrum labarum* Rosenblatt & Johnson, 1974	R	TEP	1, 9, CPUM, SIO	
*Diplectrum macropoma* (Günther, 1864)	R	TEP	1, CPUM, SIO	
*Diplectrum pacificum* Meek & Hildebrand, 1925	R	TEP	2, 4, 6, 9, CPUM	
*Diplectrum rostrum Bortone, 1974	PD	TEP	CPUM	A
*Epinephelus analogus* Gill, 1863	R	SP, TEP	1, 2, 4, 6, 9, CPUM, ICMYL, CNPE-IBUNAM	C
*Epinephelus labriformis* (Jenyns, 1840)	PM, R	TEP	1, 2, 4, 6, 9, CPUM	C
*Hyporthodus acanthistius* (Gilbert, 1892)	R	TEP	1, 2, 4, 9, CPUM, ICMYL	C
*Hyporthodus exsul (Fowler, 1944)		TEP	SIO	
*Hyporthodus niphobles* Gilbert & Starks, 1897	R	SP, TEP	1, CPUM	C
*Paralabrax loro Walford, 1936	R	TEP	9, CPUM	C
*Paranthias colonus* (Valenciennes, 1846)	R	TEP	2, 6, CPUM, ICMYL	C
*Pseudogramma thaumasia (Gilbert, 1900)	R	TEP	CPUM	
*Rypticus bicolor* Valenciennes, 1846	PM, R	TEP	1, CPUM	A
*Rypticus nigripinnis Gill, 1861		TEP	9, ICMYL	
*Serranus psittacinus* Valenciennes, 1846		TEP	2	
**FAMILY PRIACANTHIDAE**				
*Heteropriacanthus cruentatus* (Lacepède, 1801)		CT	2, 6	
*Pristigenys serrula* (Gilbert, 1891)	D	TEP	1, 2, 6, 9, CPUM	
**FAMILY APOGONIDAE**				
*Apogon pacificus* (Herre, 1935)	R	TEP	1, 6, CPUM-photo, ICMYL	
*Apogon retrosella* (Gill, 1862)	PM, R	TEP	2, 6, CPUM	
**FAMILY ECHENEIDAE**				
*Phtheirichthys lineatus (Menzies, 1791)		CT	ANSP	
*Remora osteochir (Cuvier, 1829)		CT	SIO	
*Remora remora* (Linnaeus, 1758)	D	CT	2, CPUM, CNPE-IBUNAM	
**FAMILY CARANGIDAE**				
*Alectis ciliaris* (Bloch, 1787)	PD	CT	1, 2, 9, CPUM	C
*Carangoides otrynter* (Jordan & Gilbert, 1883)	R	SP, TEP	1, 2, 4, CPUM, ICMYL	C
*Carangoides vinctus* Jordan & Gilbert, 1882	D	SP, TEP	1, 2, 9, CPUM, ICMYL, CNPE-IBUNAM	C
*Caranx caballus* Günther, 1868	PM, R	SP, TEP	1, 2, 4, 6, CPUM, ICMYL	C
*Caranx caninus* Günther, 1867	R, E	SP, TEP	1, 2, 9, 12, CPUM, SIO, CIBNOR	C
*Caranx lugubris* Poey, 1860	PD	TEP	2, CPUM	C
*Caranx melampygus* Cuvier, 1833		CT	2, 6	
*Caranx sexfasciatus* Quoy & Gaimard, 1825	R, E	AP	2, 7, 8, CPUM	C
*Chloroscombrus orqueta* Jordan & Gilbert, 1883	PM	SP, TEP	1, 2, 9, CPUM, CNPE-IBUNAM	A
*Decapterus macrosoma Bleeker, 1851	PD	AP	CPUM	C
*Decapterus muroadsi* (Temminck & Schlegel, 1844)		CT	1, 2	
*Elagatis bipinnulata* (Quoy & Gaimard, 1825)	PD	CT	1, 2, CPUM	C
*Gnathanodon speciosus* (Forsskål, 1775)		AP	2	
*Hemicaranx leucurus* (Günther, 1864)		TEP	2	
*Hemicaranx zelotes* Gilbert, 1898		TEP	2, 7	
*Oligoplites altus* (Günther, 1868)	PD	TEP	2, CPUM	C
*Oligoplites refulgens* Gilbert & Starks, 1904	PD	TEP	2, CPUM	C
*Oligoplites saurus* (Bloch & Schneider, 1801)	E	TEP	2, CPUM	
*Selar crumenophthalmus* (Bloch, 1793)	R, E	CT	1, 2, 8, CPUM, SIO	C
*Selene brevoortii* (Gill, 1863)	R, E	SP, TEP	1, 2, CPUM	A
*Selene peruviana* (Guichenot, 1866)	R	TEP	1, 2, 9, CPUM	A
*Seriola peruana* Steindachner, 1881	R	TEP	2, CPUM	C
*Seriola rivoliana* Valenciennes, 1833	PD	CT	2, CPUM	C
*Trachinotus kennedyi Steindachner, 1876	E	TEP	2, CPUM, CNPE-IBUNAM	
*Trachinotus paitensis* Cuvier, 1832		TEP	1, 2	
*Trachinotus rhodopus* Gill, 1863	PM, R, E	SP, MP, PP	1, 2, 4, 6, 7, 12, CPUM, ICMYL	
**FAMILY NEMATISTIIDAE**				
*Nematistius pectoralis* Gill, 1862	PD	TEP	2, 4, CPUM	C
**FAMILY CORYPHAENIDAE**				
*Coryphaena equiselis Linnaeus, 1758		CT	SIO	
*Coryphaena hippurus* Linnaeus, 1758	PD	CT	2, 4, CPUM-photo	C
**FAMILY LUTJANIDAE**				
*Hoplopagrus guentherii* Gill, 1862	R	SP, TEP	1, 2, 6, CPUM	C
*Lutjanus aratus* (Günther, 1864)		TEP	1	
*Lutjanus argentiventris* (Peters, 1869)	PM, R, E	SP, TEP	1, 2, 6, 12, CPUM, CNPE-IBUNAM	C
*Lutjanus colorado* Jordan & Gilbert, 1882	R, E	SP, TEP	1, 2,4, 6, 8, CPUM	C
*Lutjanus guttatus* (Steindachner, 1869)	R	TEP	1, 2, 4, 6, CPUM, SIO, ICMYL	C
*Lutjanus inermis* (Peters, 1869)	R	TEP	1, 2, CPUM, ICMYL	C
*Lutjanus jordani (Gilbert, 1898)		TEP	6	
*Lutjanus novemfasciatus* Gill, 1862	PM, R, E	SP, TEP	1, 2, 4, 6, 7, 8, 10, 12, 13, CPUM	C
*Lutjanus peru* (Nichols & Murphy, 1922)	R	SP, TEP	1, 2, 4, 9, CPUM, ICMYL	C
*Lutjanus viridis (Valenciennes, 1846)	PD	TEP	6, CPUM	
**FAMILY LOBOTIDAE**				
*Lobotes pacificus* Gilbert, 1898	PD	CT	1, CPUM	C
**FAMILY GERREIDAE**				
*Deckerichthys aureolus* (Jordan & Gilbert, 1882)	D	TEP	1, 2, 9, CPUM, ICMYL	C
*Diapterus brevirostris* (Sauvage, 1879)	E	TEP	1, 2, CPUM	C
*Eucinostomus currani* Zahuranec, 1980	PM, E	SP, TEP	1, 2, 7, 8, 9, 10, 12, 13, CPUM, CNPE-IBUNAM	C
*Eucinostomus dowii* (Gill, 1863)		SP, TEP	1	
*Eucinostomus entomelas Zahuranec, 1980		SP, TEP	CNPE-IBUNAM	
*Eucinostomus gracilis* (Gill, 1862)		TEP	1, 2, 9	
*Eugerres axillaris (Günther, 1864)	R, E	TEP	CPUM	
*Eugerres brevimanus* (Günther, 1864)		MP, PP	1	
*Eugerres lineatus (Humboldt, 1821)	E	TEP	CPUM	
*Gerres simillimus* Reagan, 1907	R, E	TEP	1, 2, 4, 7, CPUM	C
**FAMILY HAEMULIDAE**				
*Anisotremus caesius (Jordan & Gilbert, 1882)	R	MP, PP	CPUM, CNPE-IBUNAM	C
*Anisotremus interruptus* (Gill, 1862)	R	SP, TEP	1, 2, 4, 6, CPUM, ICMYL, CNPE-IBUNAM	C
*Anisotremus taeniatus* Gill, 1861	R	SP, TEP	2, 6, CPUM	C
*Conodon serrifer* Jordan & Gilbert, 1882		SP, TEP	1, ICMYL	
*Genyatremus dovii* (Günther, 1864)	R	TEP	2, CPUM, ICMYL, CNPE-IBUNAM	C
*Genyatremus pacifici* (Günther, 1864)		MP, PP	2	
*Haemulon californiensis* (Steindachner, 1876)		TEP	2	
*Haemulon flaviguttatum* Gill, 1862	R	SP, TEP	1, 2, 4, 6, CPUM, ICMYL	C
*Haemulon maculicauda* (Gill, 1862)	R	SP, TEP	2, 6, CPUM, ICMYL	C
*Haemulon scudderii* Gill, 1862	R	SP, TEP	1, 2, CPUM	C
*Haemulon sexfasciatum* Gill, 1862	R	TEP	1, 2, 6, CPUM	C
*Haemulon steindachneri* (Jordan & Gilbert, 1882)	R	AA	2, CPUM, ICMYL, CNPE-IBUNAM	C
*Haemulopsis axillaris* (Steindachner, 1869)	R	MP, PP	2, CPUM	C
*Haemulopsis elongatus* (Steindachner, 1879)	R	MP, PP	1, CPUM, ICMYL	C
*Haemulopsis leuciscus* (Günther, 1864)	E	TEP	1, 2, 6, 9, CPUM, CNPE-IBUNAM	C
*Haemulopsis nitidus* (Steindachner, 1869)	R	TEP	1, CPUM	
*Microlepidotus brevipinnis (Steindachner, 1869)	R	TEP	CPUM	C
*Orthopristis chalceus* (Günther, 1864)	R	TEP	2, 4, CPUM	
*Orthopristis reddingi* Jordan & Richardson, 1895	R	TEP	1, 2, CPUM	
*Pomadasys bayanus* Jordan & Evermann, 1898	E	TEP	1, 2, CPUM	
*Pomadasys branickii* (Steindachner, 1879)	E	TEP	1, 7, 10, CPUM, ICMYL	
*Pomadasys macracanthus* (Günther, 1864)	E	TEP	2, CPUM	
*Pomadasys panamensis* (Steindachner, 1876)		TEP	1, 2, 9, CNPE-IBUNAM	C
*Xenichthys xanti* Gill, 1863	R	SP, TEP	2, CPUM, CNPE-IBUNAM	C
**FAMILY SPARIDAE**				
*Calamus brachysomus* (Lockington, 1880)	R	SP, TEP	1, 2, CPUM	C
**FAMILY SCIAENIDAE**				
*Bairdiella armata* Gill, 1863		TEP	1, 2	
*Bairdiella ensifera* (Jordan & Gilbert, 1882)		MP, PP	1, 2	
*Bairdiella icistia* (Jordan & Gilbert, 1882)		CP, MP	2	
*Corvula macrops (Steindachner, 1876)	R	TEP	CPUM	
*Cynoscion nannus* Castro-Aguirre & Arvizu-Martínez, 1976	PD	CP, MP	1, CPUM	C
*Cynoscion phoxocephalus* Jordan & Gilbert, 1882		MP, PP	1, 9, CPUM, CIBNOR	C
*Cynoscion reticulatus* (Günther, 1864)		TEP	1, 2	
*Cynoscion stolzmanni (Steindachner, 1879)		TEP	ICMYL	
*Elattarchus archidium* (Jordan & Gilbert, 1882)	PD	TEP	2, CPUM	C
*Isopisthus remifer* Jordan & Gilbert, 1882		TEP	2	
*Larimus acclivis* Jordan & Bristol, 1898	PD	TEP	2, CPUM	C
*Larimus argenteus* (Gill, 1863)	PD	TEP	1, 2, CPUM, ICMYL	C
*Larimus effulgens* Gilbert, 1898	PD	TEP	1, 2, 9, CPUM	C
*Menticirrhus elongatus* (Günther, 1864)	E	SP, TEP	2, CPUM	C
*Menticirrhus nasus* (Günther, 1868)		TEP	2, CPUM, CNPE-IBUNAM	C
*Menticirrhus panamensis* (Steindachner, 1875)		TEP	1, 2	
*Menticirrhus undulatus* (Girard, 1854)	PD	SP, TEP	1, CPUM	C
*Micropogonias altipinnis* (Günther, 1864)		TEP	2	
*Micropogonias ectenes (Jordan & Gilbert, 1882)	PD	SP, TEP	CPUM	C
*Micropogonias megalops* (Jordan & Gilbert, 1884)		CP, MP	1	
*Odontoscion xanthops* Gilbert, 1898	R	TEP	2, CPUM	
*Ophioscion imiceps* (Jordan & Gilbert, 1882)		MP, PP	2, ICMYL, CNPE-IBUNAM	
*Ophioscion scierus* (Jordan & Gilbert, 1884)		MP, PP	2, ICMYL	
*Ophioscion strabo Gilbert, 1897		TEP	ICMYL	
*Ophioscion typicus Gill, 1863		TEP	CNPE-IBUNAM	
*Ophioscion vermicularis (Günther, 1867)	PD	MP, PP	CPUM	C
*Pareques fuscovittatus (Kendall & Radcliffe, 1912)	R	MP	6, CPUM	
*Umbrina bussingi* López S., 1980	PD	MP, PP	1, 9, CPUM, ICMYL	C
*Umbrina dorsalis* Gill, 1862	R, E	TEP	2, 12, CPUM	
*Umbrina xanti* Gill, 1862	R, E	SP, TEP	1, 2, CPUM	C
**FAMILY POLYNEMIDAE**				
*Polydactylus approximans* (Lay & Bennett, 1839)	E	TEP	1, 2, 9, CPUM, SIO, CNPE-IBUNAM	C
*Polydactylus opercularis* (Gill, 1863)	E	SP, TEP	1, 2, 8, CPUM	C
**FAMILY MULLIDAE**				
*Mulloidichthys dentatus* (Gill, 1862)	R	TEP	2, 6, CPUM, ICMYL	C
*Pseudupeneus grandisquamis* (Gill, 1863)	R	TEP	1, 2, 9, CPUM, CNPE-IBUNAM	C
**FAMILY KYPHOSIDAE**				
*Kyphosus analogus* (Gill, 1862)	R	TEP	2, 4, CPUM	C
*Kyphosus elegans* (Peters, 1869)	R	TEP	2, 4, 5, CPUM	C
*Kyphosus ocyurus* (Jordan & Gilbert, 1882)	R	CT	1, 2, CPUM, SIO, ICMYL	C
**FAMILY CHAETODONTIDAE**				
*Chaetodon humeralis* Günther, 1860	PM, R	TEP	1, 2, 4, 5, 6, 9, CPUM	
*Johnrandallia nigrirostris* (Gill, 1862)	PM, R	SP, TEP	2, 6, CPUM	I
**FAMILY POMACANTHIDAE**				
*Holacanthus passer* Valenciennes, 1846	R	TEP	2, 6, CPUM	
*Pomacanthus zonipectus* (Gill, 1862)	R	SP, TEP	1, 2, 6, 9, CPUM, ICMYL	
**FAMILY CIRRHITIDAE**				
*Cirrhitichthys oxycephalus* (Bleeker, 1855)	R	TEP	2, 6, CPUM	
*Cirrhitus rivulatus* Valenciennes, 1846	PM, R	TEP	2, 4, 5, 6, CPUM	
**FAMILY MUGILIDAE**				
*Agonostomus monticola* (Bancroft, 1882)	E	AA	1, 3, 10, 12, 13, CPUM, ICMYL	
*Chaenomugil proboscideus (Günther, 1861)	PM	TEP	CPUM, ICMYL	
*Mugil cephalus* Linneaus, 1758		CT	1, 2	
*Mugil curema* Valenciennes, 1836	PM, E	AA	2, 3, 4, 5, 7, 8, 10, 12, 13, CPUM	C
**FAMILY POMACENTRIDAE**				
*Abudefduf declivifrons (Gill, 1862)	PM, R	TEP	4, 5, CPUM	
*Abudefduf troschelii* (Gill, 1862)	PM, R	SP, TEP	2, 5, 6, CPUM	
*Chromis atrilobata* Gill, 1862	R	TEP	2, 6, CPUM	
*Microspathodon bairdii* (Gill, 1862)	PM, R	TEP	2, 5, CPUM	
*Microspathodon dorsalis* (Gill, 1862)	PM, R	SP, TEP	2, 4, 5, 6, CPUM	I
*Stegastes acapulcoensis* (Fowler, 1944)	PM, R	MP, PP	2, 5, 6, CPUM	
*Stegastes flavilatus* (Gill, 1862)	PM, R	MP, PP	2, 5, 6, CPUM, ICMYL	
*Stegastes rectifraenum* (Gill, 1862)	PM, R	SP, CP, MP	2, 5, CPUM, ICMYL	
**FAMILY LABRIDAE**				
*Bodianus diplotaenia* (Gill, 1862)	R	SP, TEP	2, 6, CPUM, ICMYL	
*Halichoeres chierchiae* di Caporiacco, 1947	R	TEP	2, 6, CPUM	
*Halichoeres dispilus* (Günther, 1864)	PM, R	TEP	2, 4, 5, 6, CPUM	
*Halichoeres nicholsi* (Jordan & Gilbert, 1882)	R	TEP	2, 6, CPUM	
*Halichoeres notospilus* (Günther, 1864)	PM, R	TEP	2, 6, CPUM	
*Iniistius pavo (Valenciennes, 1840)	R	AP	CPUM	
*Novaculichthys taeniourus (Lacepède, 1801)	R	AP	6	
*Thalassoma grammaticum Gilbert, 1890	R	TEP	6, CPUM	
*Thalassoma lucasanum* (Gill, 1862)	PM, R	TEP	2, 5, 6, CPUM	
**FAMILY SCARIDAE**				
*Calotomus carolinus (Valenciennes, 1840)	PD	CT	6, CPUM	
*Nicholsina denticulata (Evermann & Radcliffe, 1917)	R	SP, TEP	CPUM	
*Scarus compressus (Osburn & Nichols, 1916)	R	TEP	6, CPUM	
*Scarus perrico* Jordan & Gilbert, 1882	R	TEP	2, 6, CPUM	C
**FAMILY URANOSCOPIDAE**				
*Astroscopus zephyreus* Gilbert & Starks, 1897	PD	SP, TEP	2, CPUM	A
**FAMILY TRIPTERYGIIDAE**				
*Axoclinus storeyae* (Brock, 1940)	PM, R	CP, MP	2, CPUM	
*Enneanectes carminalis* Jordan & Gilbert, 1882		TEP	2	
*Enneanectes glendae Rosemblatt, Miller &Hastings, 2013	R	CP, MP	CPUM	
*Enneanectes macrops Rosemblatt, Miller &Hastings, 2013	R	MP	CPUM	
**FAMILY LABRISOMIDAE**				
*Brockius striatus* (Hubbs, 1953)	PM, R	CP, MP	2, CPUM	
*Labrisomus multiporosus Hubbs, 1953	PM, R	TEP	CPUM	
*Labrisomus xanti Gill, 1860		SP, TEP	ICMYL	
*Malacoctenus ebisui* Springer, 1959	R	TEP	2, CPUM	
*Malacoctenus hubbsi* Springer, 1959	R	TEP	2, 5, CPUM	
*Malacoctenus mexicanus Springer, 1959	R	TEP	CPUM	
*Malacoctenus tetranemus (Cope, 1877)	PM, R	TEP	CPUM	
*Malacoctenus zonifer (Jordan & Gilbert, 1882)	PM	TEP	CPUM	
*Paraclinus mexicanus (Gilbert, 1904)	PM	TEP	CPUM	
*Starksia posthon Rosenblatt & Taylor, 1971	R	MP, PP	CPUM	
*Starksia spinipenis (Al-Uthman, 1960)	R	CP, MP	CPUM	
**FAMILY CHAENOPSIDAE**				
*Acanthemblemaria balanorum* Brock, 1940		TEP	2	
*Acanthemblemaria macrospilus Brock, 1940	R	CP, MP	CPUM	
*Coralliozetus boehlkei Stephens, 1963	R	TEP	CPUM	
*Ekemblemaria myersi Stephens, 1963	R	TEP	CPUM	
*Protemblemaria bicirrus (Hildebrand, 1946)	R	TEP	CPUM	
**FAMILY DACTYLOSCOPIDAE**				
*Dactyloscopus amnis* Miller & Briggs, 1962	E	MP, PP	1, 12, CPUM	
**FAMILY BLENNIIDAE**				
*Entomacrodus chiostictus (Jordan & Gilbert, 1882)	PM	TEP	CPUM	
*Hypsoblennius brevipinnis (Günther, 1861)	R	TEP	CPUM, SIO, ICMYL	
*Ophioblennius steindachneri* Jordan & Evermann, 1898	PM, R	TEP	2, 5, 6, CPUM, ICMYL	
*Plagiotremus azaleus* (Jordan & Bollman, 1890)	R	TEP	2, 6, CPUM	
**FAMILY ELEOTRIDAE**				
*Dormitator latifrons* (Richardson, 1844)	E	SP, TEP	1, 7, 8, 10, 12, 13, CPUM, CNPE-IBUNAM	
*Eleotris picta* Kner, 1863	E	TEP	1, 7, 8, 10, 12, 13, CPUM, CNPE-IBUNAM	
*Gobiomorus maculatus* (Günther, 1859)	E	TEP	1, 2, 7, 8, 10, 12, 13, CPUM, CNPE-IBUNAM	
*Gobiomorus polylepis Ginsburg, 1953	E	Brackish	CPUM	
**FAMILY GOBIIDAE**				
*Awaous banana* (Valenciennes, 1837)	E	SP, TEP	3, 8, 12, CPUM	
*Barbulifer mexicanus* Hoese & Larson, 1985		CP, MP	1	
*Bathygobius andrei (Sauvage, 1880)	E	MP, PP	CPUM	
*Bathygobius ramosus* Ginsburg, 1947	PM	SP, TEP	1, 2, 5, CPUM, ICMYL	
*Bollmannia marginalis Ginsburg, 1939		TEP	9	
*Bollmannia stigmatura Gilbert, 1892		TEP	ICMYL	
*Coryphopterus urospilus Ginsburg, 1938	R	TEP	CPUM	
*Ctenogobius sagittula* (Günther, 1861)	E	TEP	12, CPUM	
*Elacatinus punticulatus (Ginsburg, 1938)	R	TEP	CPUM	
*Gobionellus microdon* (Gilbert, 1892)	E	TEP	1, 3, 7, 10, 12, 13, CPUM	
*Gymneleotris seminuda (Günther, 1864)	R	TEP	CPUM	
*Microgobius miraflorensis* Gilbert & Starks, 1904		TEP	1	
*Sicydium multipunctatum Regan, 1905	E	Freshwater	CPUM	
**FAMILY MICRODESMIDAE**				
*Clarkichthys bilineatus* (Clark, 1936)	PM	TEP	CPUM	
*Microdesmus dorsipunctatus* Dawson, 1968	E	TEP	12, CPUM	
**FAMILY EPHIPPIDAE**				
*Chaetodipterus zonatus* (Girard, 1858)	R	SP, TEP	1, 3, 8, 9, CPUM	C
*Parapsettus panamensis* Steindachner, 1876		TEP	1, 3	
**FAMILY ZANCLIDAE**				
*Zanclus cornutus (Linnaeus, 1758)	R	SP, TEP	6, CPUM	
**FAMILY ACANTHURIDAE**				
*Acanthurus triostegus* (Linnaeus, 1758)	PM, R	AP	2, 5, 6, CPUM	
*Acanthurus xanthopterus* Valenciennes, 1835	R	AP	2, 6, CPUM	A
*Prionurus punctatus* Gill, 1862	PM, R	TEP	2, 5, 6, CPUM	A
**FAMILY SPHYRAENIDAE**				
*Sphyraena ensis* Jordan & Gilbert, 1882	R	TEP	1, 2, CPUM	C
**FAMILY TRICHIURIDAE**				
*Trichiurus nitens Garman, 1899	PD	CT	CPUM	I
**FAMILY SCOMBRIDAE**				
*Auxis brachydorax* Collette & Aadland 1996		SP, TEP	2	
*Euthynnus lineatus* Kishinouye, 1920	PD	SP, TEP	1, 2, CPUM, SIO	C
*Katsuwonus pelamis* (Linnaeus, 1758)		CT	2	
*Sarda orientalis (Temminck & Schlegel, 1844)	PD	AP	CPUM	C
*Scomberomorus sierra* Jordan & Starks, 1895	PD	SP, TEP	1, 2, CPUM	C
*Scomber japonicus Houttuyn, 1782	PD	CT	CPUM	C
*Thunnus alalunga* (Bonnaterre, 1788)		CT	1	
**FAMILY ISTIOPHORIDAE**				
*Istiophorus platypterus* (Shaw, 1792)		AP	2	
**FAMILY STROMATEIDAE**				
*Peprilus medius* (Peters, 1869)	PD	TEP	2, CPUM	C
*Peprilus snyderi Gilbert & Starks, 1904	PD	SP, TEP	CPUM	C
**ORDER PLEURONECTIFORMES**				
**FAMILY PARALICHTHYIDAE**				
*Ancylopsetta dendrítica* Gilbert, 1890	PD	TEP	1, 2, 9, CPUM	C
*Citharichthys gilberti* Jenkins & Evermann, 1889	E	SP, TEP	1, 8, 12, CPUM	
*Citharichthys platophrys Gilbert, 1891		TEP	SIO	
*Cyclopsetta panamensis* (Steindachner, 1876)		TEP	1	
*Cyclopsetta querna* (Jordan & Bollman, 1890)	PD	TEP	1, 2, 9, CPUM, SIO	C
*Etropus crossotus* Jordan & Gilbert, 1882	PD	SP, TEP	1, 2, 9, CPUM, SIO	C
*Etropus ectenes Jordan, 1889	PD	SP, TEP	CPUM, SIO	
*Etropus peruvianus* Hildebrand, 1946		TEP	1	
*Hippoglossina tetrophthalma* (Gilbert, 1890)		TEP	2	
*Paralichthys woolmani* Jordan & Williams, 1897	D	TEP	1, 2, 9, CPUM, ICMYL	C
*Syacium latifrons* (Jordan & Gilbert, 1882)	PD	SP, TEP	1, 9, CPUM	C
*Syacium longidorsale Murakami & Amaoka, 1992		PP	CIBNOR	
*Syacium ovale* (Günther, 1864)	PD	TEP	1, 2, 9, CPUM, ICMYL	C
**FAMILY BOTHIDAE**				
*Bothus constellatus* (Jordan, 1889)	PD	TEP	1, 2, 9, CPUM	A
*Bothus leopardinus (Günther, 1862)		TEP	9	
*Engyophrys sanctilaurentii* Jordan & Bollman, 1890		TEP	1, SIO	
**FAMILY ACHIRIDAE**				
*Achirus klunzingeri* (Steindachner, 1879)	E	MP, PP	12, CPUM	
*Achirus mazatlanus* (Steindachner, 1869)	E	TEP	1, 7, 12, CPUM	
*Achirus scutum* (Günther, 1862)	E	TEP	1, CPUM	
*Trinectes fonsecensis* (Günther, 1862)	E	TEP	1, 9, 10, 12, 13, CPUM	I
**FAMILY CYNOGLOSSIDAE**				
*Symphurus atricaudus* (Jordan & Gilbert, 1880)		CP, MP	1	
*Symphurus callopterus Munroe & Mahadeva, 1989		TEP	CAS	
*Symphurus elongatus* (Günther, 1868)	PD	SP, TEP	1, CPUM	I
*Symphurus leei Jordan & Bollman, 1890		MP, PP	9, SIO, ICMYL	
**ORDER TETRADONTIFORMES**				
**FAMILY BALISTIDAE**				
*Balistes polylepis* Steindachner, 1876	R	SP, TEP	1, 2, 4, 6, 9, CPUM	
*Canthidermis maculata (Bloch, 1786)	R	CT	CPUM	
*Pseudobalistes naufragium* (Jordan & Starks, 1895)	R	SP, TEP	2, 8, CPUM	
*Sufflamen fraenatum* (Latreille, 1804)	PD	AP	11, CPUM	
*Sufflamen verres* (Gilbert & Starks, 1904)	R	SP, TEP	2, 4, 6, CPUM	C
**FAMILY MONACANTHIDAE**				
*Aluterus monoceros (Linnaeus, 1758)	R	CT	9, CPUM	C
*Aluterus scriptus* (Osbeck, 1765)	R	CT	1, 6, CPUM- photo	
**FAMILY OSTRACIIDAE**				
*Ostracion meleagris* Shaw, 1796	R	AP	2, 6, CPUM	
**FAMILY TETRAODONTIDAE**				
*Arothron hispidus (Linnaeus, 1758)	R	CT	CPUM, ICMYL	
*Arothron meleagris* (Lacèpede, 1798)	R	CT	2, 6, CPUM	I
**Canthigaster janthinoptera (Bleeker, 1855)	R	AP	CPUM	
*Canthigaster punctatissima* (Günther, 1870)	R	TEP	2, 6, CPUM	
*Lagocephalus lagocephalus (Linnaeus, 1758)		CT	9	
*Sphoeroides sechurae Hildebrand, 1946	R	TEP	9, CPUM	A
*Sphoeroides annulatus* (Jenyns, 1842)	R, E	SP, TEP	1, 2, 4, 6, 8, 12, CPUM, SIO	
*Sphoeroides lobatus* (Steindachner, 1870)	R	TEP	2, 6, CPUM	I
**FAMILY DIODONTIDAE**				
*Diodon holocanthus* Linnaeus, 1758	R	CT	2, 6, 8, 9, CPUM	
*Diodon hystrix* Linnaeus, 1758	R	CT	1, 2, 4, 6, 9, CPUM	I

* New record for the state of Michoacan. **New record for the Mexico. Collection habitat: Reef (R), rocky intertidal or tidal pool (PM), estuary (E), demersal (D)
pelagic-demersal (PD). Zoogeographical affinity: Circumtropical (CT), Amphiamerican (AA), Transpacific (AP)
San Diegan province (SP), Cortés province (CP), Mexican province (MP), Panamic province (PP). Record from literature: Castro-Aguirre et al. 2006 (1), Madrid-Vera et al. 1998 (2), Medina-Nava et al. 2005 (3), Galván-Torres 1989 (4), Aguirre-Villaseñor 1991 (5), Domínguez-Domínguez 1998 (6), González-Luna 2000 (7), Madrigal-Guridi 2006 (8), Sánchez-Aguilar 2007 (9), Sandoval-Huerta et al. 2012 (10), Palacios-Morales et al. 2014 (11), Sandoval-Huerta et al. 2014 (12), Sandoval-Huerta et al. 2015 (13). Records from Fish Collection of Universidad Michoacana de San Nicolás de Hidalgo
(CPUM), Fish Collection of the Institute of Biology of the National Autonomous University of Mexico (CNPE-IBUNA), fish collection of Instituto de Ciencias del Mar y Limnología (ICMYL), fish collection of California Academy of Sciences, San Francisco California, E.U.A. (CAS), the Marine Vertebrate Collection of Scrippps Institution of Oceanography, San Diego, California (SIO) and fish collection of Biological Research Center Northwest S.C. (CIBNOR). Fishery importance: personal consumption (A), commercial use (C) and discarded (I).

Of the total identified species, 69% were collected and deposited at the CPUM, 22% were obtained from literature records, 7.5% from the review of museum specimens, and 1% from databases of ichthyological collections (Table 1). In addition, seven species were recorded through video and photographic evidence: *Ginglymostoma unami* Del Moral Flores, Ramírez-Antonio, Angulo y Pérez-Ponce de León, 2015, *Pristis pristis* (Linnaeus, 1758), *Aetobatus laticeps* (Euphrasen, 1790), *Apogon pacificus* (Herre, 1935), *Coryphaena hippurus* Linnaeus, 1758, *Kyphosus ocyurus* (Jordan & Gilbert, 1882), and *Novaculichthys taeniourus* (Lacepède, 1801). Of the 436 species, 131 were new records for the Michoacán State, and *Canthigaster janthinoptera* (Bleeker, 1855) was a new record for Mexico (Table 1).

**Figure 2.**
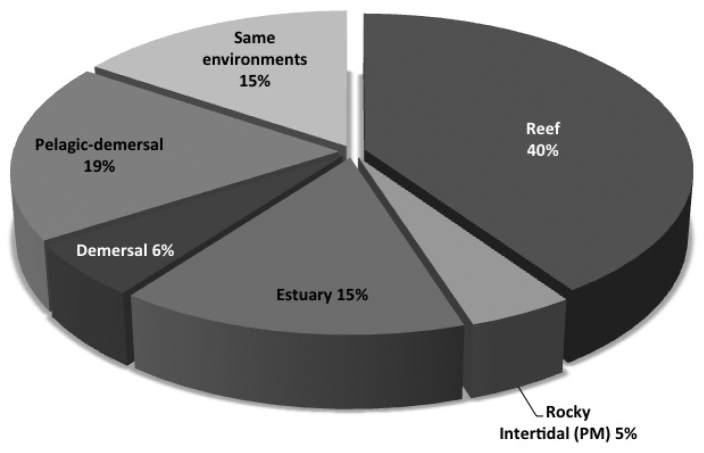
Percent of species collected in each habitat.

For the specimens collected during field trips, some were collected from a single habitat type: 123 (40%) were collected in reefs, 57 (19%) in the pelagic-demersal zone, 46 (15%) in estuarine zones, 17 (6%) in the demersal zones, and 14 (5%) in rocky intertidal zones. Forty-seven species were collected in more than one habitat type (Table 1 and Fig. 2).

**Figure 3.**
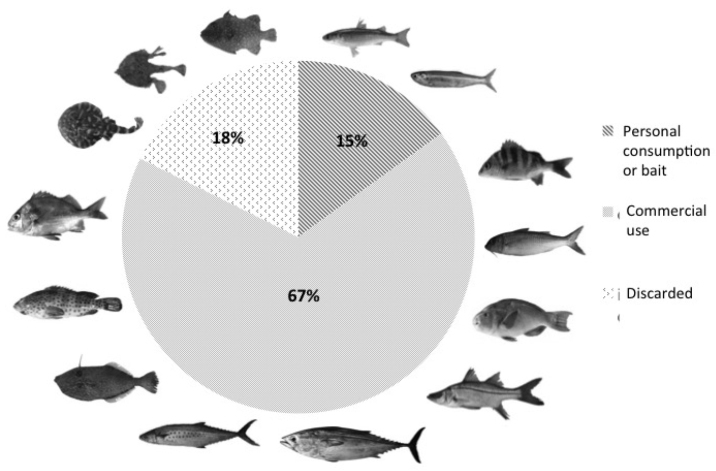
Classification of commercial importance of the species captured in the Michoacán coastal fisheries.

The artisanal fishery captures yielded 154 species. The families with the highest number of species were Carangidae (17), Haemulidae (15), Sciaenidae (13), Serranidae (10), and Lutjanidae (7). Of these, 104 (68%) were commercially valuable, 23 (15%) were used for direct consumption or as bait. Twenty-seven (18%) were bycatch, that are normally rejected and thrown back (Fig. 3).

**Figure 4.**
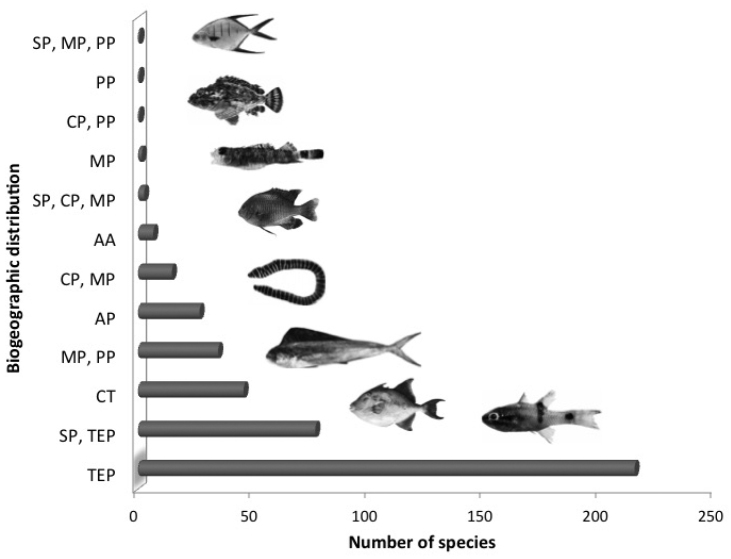
Biogeographic affinity of the fish fauna recorded on the Michoacán Coast. TEP, Tropical Eastern Pacific; CT, Circumtropical; AP, Transpacific species; AA, Amphi-American species; SP, San Diegan province; CP, Cortez province; MP, Mexican province; PP, Panamic province.

Forty-six (11%) of the species were circumtropical, 27 (6%) transpacific, and seven (2%) amphi-American; whereas 350 (81%) belonged to the TEP. Of these 77 (18%) were widely distributed from the San Diegan province to the Panamic province, and 3 (1%) from the San Diegan province to the Mexican province. The largest number of species, 216 (49.2%), were found in the three provinces of the TEP; 15 (3%) were restricted to the Cortez and the Mexican provinces; 35 (8%) were limited to the Mexican and Panamic provinces; and 2 (0.5%) were endemic to the Mexican province (Fig. 4). Six species collected in the estuarine ecosystems occurred in fresh or brackish water habitats and were not included in the marine biogeographical affinity categories: *Gobiomorus polylepis* Ginsburg, 1953 (brackish), *Sicydium multipunctatum* Regan, 1905, *Gobiesox mexicanus* Briggs & Miller, 1960, and *Poecilia butleri* Jordan, 1889 (fresh water). The introduced species *Pterygoplichthys disjuntivus* (Weber, 1991) and *Cyprinus carpio* Linnaeus, 1758 were also omitted.

## Discussion

The present checklist represents the most updated and comprehensive systematic list of fishes recorded from the coast of the Mexican State of Michoacán. Of the species cataloged, 30% were first records for this State. The highest proportion of cataloged species was collected in reefs (40%). The pelagic zone accounted for 24% of the species collected, indicated a requirement for future studies of the demersal and pelagic zones with increased sampling effort (Fig. 2). The highest number of new records for Michoacán was found in reefs, chiefly species exhibiting cryptic behavior (Table 1). This could be related to the sampling methods employed, which had not been previously used; the few previously reported species with cryptic behavior were primarily bycatch (Madrid-Vera 1998, Castro-Aguirre et al. 2006, Moncayo-Estrada et al. 2006, Chávez-Comparan 2008, Márquez-Espinoza 2012). Another source of new species records from Michoacán was the intertidal zone (Table 1), for which no published records are available. In general, the number of species in the area may be increased if sampling effort is expanded and records from shrimp and tuna bycatches are included. A new record was obtained for Mexico, two specimens of *Canthigaster janthinoptera* (Bleeker, 1855) were collected from Barco Hundido del Faro de Bucerias (CPUM 4532, N 18°21'8.82'W -103°31'18.71'), which identification was corroborated by BLAST (http://www.ncbi.nlm.nih.gov/genbank) and by boldsystems (http://www.boldsystems.org), searches showing 99% similarity in the cytochrome oxidase subunit 1 gene (COX-1) to specimens identified as *Canthigaster janthinoptera* from the south Tropical Eastern Pacific and the Indo-Pacific Ocean (GenBank accession numbers: KX505745 and KX505746). One specimen of *Bathygobius andrei* (Sauvage, 1880) (Gobiidae) was also reported in the Chuta estuary (CPUM 3296, 18°2'1"N and 102°33'33"W), representing an extension of its previously known northern distribution limit of the coast of Chiapas (Gómez-González et al. 2012). One specimen of *Calotomus carolinus* (Valenciennes, 1840) was collected from rocky reef in Faro de Bucerias location (18°20'50"N and 103°30'37"W), extending its extension range in the TEP.

In artisanal fishing (Fig. 3), the species considered to have the highest economic value belong chiefly to Carangidae (e.g. *Alectis ciliaris*, (Bloch, 1787)), Lutjanidae (e.g. *Lutjanus guttatus*, (Steindachner, 1869)), Paralichthyidae (e.g. *Cyclopsetta querna*, (Jordan & Bollman, 1890)), Centropomidae (e.g. *Centropomus armatus*, Gill, 1863), and Serranidae (e.g. *Epinephelus labriformis*, (Jenyns, 1840)) (Table 1). Most of the elasmobranch capture, with the exception of the fins, is considered of low economic value. A high number of neonatal and juvenile hammerhead sharks (*Sphyrna* sp.), were captured, as well as pregnant *Rhinobatos glaucostigma* Jordan & Gilbert, 1883, *Gymnura marmorata* (Cooper, 1863), and *Urotrygon* spp. In general, the elasmobranchs, due to their unique biological and ecological characteristics, present low population growth and are considered highly vulnerable (Frisk et al. 2005, Hutchings et al. 2012). We accordingly recommend review and enforcement of the relevant legislation.

Bycatch in commercial fishing is frequently used for personal consumption, bait (~50%), or discarded (Fig. 3). Species with no current market value may have high nutrient value; hence the number of species with potential to be commercialized is underestimated. In offshore fisheries, these species often have commercial value. For instance, *Scorpaena mystes* (Jordan & Starks, 1895) reaches 35.6 cm and is marketed in regions such as Baja California. *Trichiurus nitens* Garman, 1899 supports a small fishery in the central portion of the littoral zone of Ecuador (pers. obs. Romero-Gallardo), whereas, in Michoacán, this species is not used for human consumption.

It was observed that 49% of the listed species are reported as also occurring throughout the three TEP provinces, with 8% of the species reported only in the Panamic and Mexican provinces, being mostly of tropical affinity, agreeing with previous fish fauna surveys in the area (Castro-Aguirre et al. 2006, Moncayo-Estrada 2006). The presence of 81 species (19%) with affinity to the San Diegan province (Fig. 4), a temperate-warm zone, reflects the dynamics of the current flow system of the Michoacán coast, reaffirming this region as a transition zone.

Although visual censuses and photo identification of fish species is widely used for the study of richness, diversity, and ecology of marine habitats (Aguilar-Palomino 2002, Palacios-Salgado 2005, Galván-Villa 2016), it is necessary to rely on reference organisms for taxonomic corroboration. A high proportion of small, nocturnal, or ecologically cryptic species may not be counted in a visual census, especially when the fish fauna of the area under study is not well known, as is the case for the Michoacán State.

The collections obtained in the present study enriched the records of the CPUM collection by 19%, since the majority of marine species previously collected remain in collections outside of Michoacán. Many species reported in this study as new records (Table 1) were included by Robertson and Allen (2015), although these authors listed them in the littoral zone of Michoacán coastline only as potential distribution based on habitat suitability. We have confirmed the distribution of such fish, as exemplified by the first formal record of 24 ecologically cryptic species. Our work expanded on the most complete fish fauna checklist previously available for the area by 32.5% (Madrid et al. 1998) and will undoubtedly represent important input for decisions about conservation and management of the coastal area of Michoacán State.
